# Microbial Polyethylene Terephthalate Hydrolases: Current and Future Perspectives

**DOI:** 10.3389/fmicb.2020.571265

**Published:** 2020-11-11

**Authors:** Clodagh M. Carr, David J. Clarke, Alan D. W. Dobson

**Affiliations:** ^1^School of Microbiology, University College Cork, Cork, Ireland; ^2^SSPC-SFI Research Centre for Pharmaceuticals, University College Cork, Cork, Ireland

**Keywords:** plastic, PET hydrolases, synthetic polymer, biorecycling, bioremediation, circular economy, cutinases, PETases

## Abstract

Plastic has rapidly transformed our world, with many aspects of human life now relying on a variety of plastic materials. Biological plastic degradation, which employs microorganisms and their degradative enzymes, has emerged as one way to address the unforeseen consequences of the waste streams that have resulted from mass plastic production. The focus of this review is microbial hydrolase enzymes which have been found to act on polyethylene terephthalate (PET) plastic. The best characterized examples are discussed together with the use of genomic and protein engineering technologies to obtain PET hydrolase enzymes for different applications. In addition, the obstacles which are currently limiting the development of efficient PET bioprocessing are presented. By continuing to study the possible mechanisms and the structural elements of key enzymes involved in microbial PET hydrolysis, and by assessing the ability of PET hydrolase enzymes to work under practical conditions, this research will help inform large-scale waste management operations. Finally, the contribution of microbial PET hydrolases in creating a potential circular PET economy will be explored. This review combines the current knowledge on enzymatic PET processing with proposed strategies for optimization and use, to help clarify the next steps in addressing pollution by PET and other plastics.

## Introduction

**FIGURE 1:**
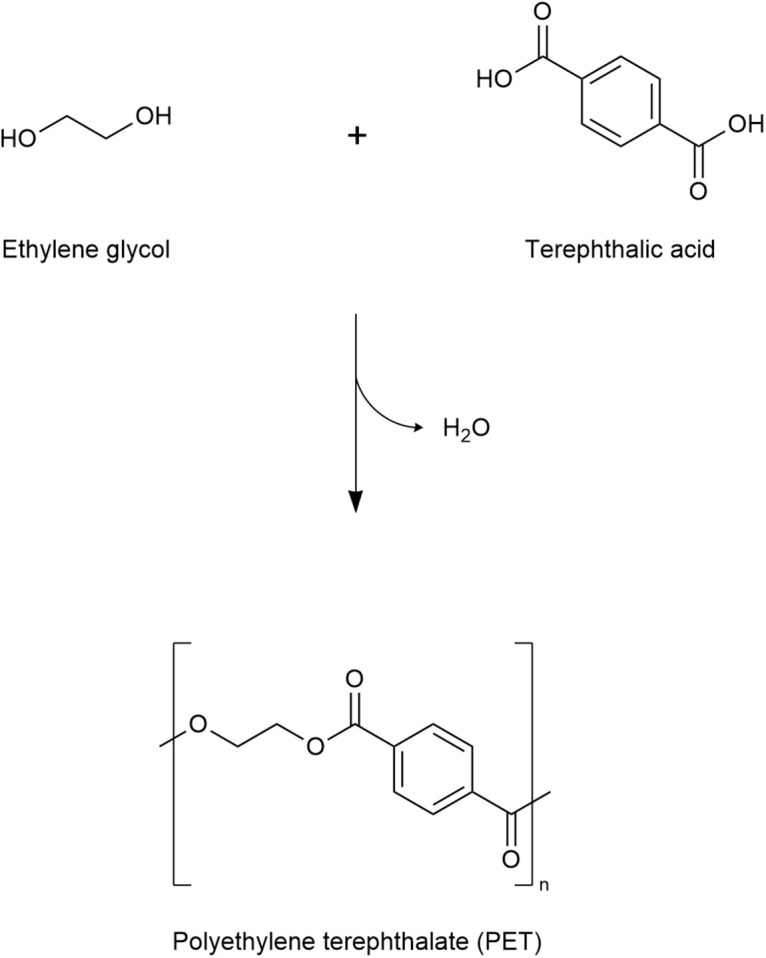
Structure of PET polymer. Polycondensation reaction of ethylene glycol (EG) and terephthalic acid (TPA) monomers gives polymeric polyethylene terephthalate (PET), with water as a byproduct.

The use of plastics has become an integral part of modern society, with annual production exceeding 350 million metric tons (Danso et al., 2019; PlasticsEurope, 2019). Polyethylene terephthalate (PET), a crude-oil derived synthetic polymer, is currently one of the most extensively used plastics (Liu et al., 2019). In 2017, PET production capacity reached over 30 million tons per annum (PlasticsInsight, 2017). Approximately 485 billion PET bottles were produced in 2016, and an estimated 583.3 billion plastic bottles are predicted to be manufactured in 2021 (Garside, 2019). This polymer consists of repeating units of terephthalic acid (TPA) and ethylene glycol (EG) (Figure 1). Due to its light weight, durability and moldability, PET is convenient both in terms of its production and its utility; where it is used in containers, films, and fibers, in addition to bottles. However, the qualities which have made plastic an attractive resource, are equally as responsible for the damage that is typically caused once it becomes waste. Mismanagement of PET-based material has resulted in its frequent disposal and accumulation in the environment where its robustness is problematic, as it is not particularly susceptible to biodegradation. The high stability of the polymer’s backbone, together with its crystallinity and surface hydrophobicity are some of the underlying factors which restrict the natural breakdown of this plastic (Kawai et al., 2019; Liu et al., 2019; Jaiswal et al., 2020).

As the primary use for plastics is packaging, PET is often used in short-term, single use applications. Despite the fact that recycling systems are in place, over half of the PET that is produced escapes collection, and instead ends up in landfills or is released into nature. Only a very limited amount of PET is recycled back into its original form, with the majority being downcycled into lower quality, non-recyclable plastics (Kawai et al., 2014). PET waste persists in both marine and terrestrial environments, which often results in harm or death to some of the inhabitants. Annually, plastic pollution is considered responsible for the death of around 1 million sea birds, as well as 100,000 marine mammals and turtles (Jaiswal et al., 2020). Furthermore, the partial breakdown of discarded plastics and treatment of synthetic fibers has resulted in the widespread shedding of microplastics and microfibers, respectively (Hiraga et al., 2019). In this form, plastics readily contaminate marine ecosystems and this has resulted in them entering the animal and human food chain, which has been linked to various adverse health effects, including immune disorders and congenital disabilities, as well as cancer (Jaiswal et al., 2020). It is now believed that soils may constitute an even larger sink for plastic, and since microplastics can readily leach into groundwater, or contaminate other water reserves, this problem is thought to affect organisms across the entire terrestrial ecosystem (Song et al., 2019). Recently, there has been a marked increase in awareness surrounding the potential detrimental environmental impact of plastics. Therefore, new solutions and technologies, which assist in managing plastic waste, are of major importance (Danso et al., 2019).

Synthetic polymers were once considered resistant to microbial degradation, however, more recent studies have shown that certain microbes produce hydrolase enzymes that enable them to either break down or modify PET. Microbes have evolved a variety of hydrolytic enzymes that allow them to degrade and process polymers that are prevalent in nature, for example the waxy plant polyester cutin. In a somewhat similar way, some microorganisms are using enzymes to take advantage of PET as an energy source, despite the significant differences between the synthetic and natural polymer structures (Danso et al., 2019; Kawai et al., 2019; Taniguchi et al., 2019).

Enzymes which display PET hydrolyzing activities include carboxylic ester hydrolase enzymes (EC 3.1.1) such as cutinases, lipases, and esterases (Guebitz and Cavaco-Paulo, 2008; Kawai et al., 2020). These hydrolases have been isolated from bacterial sources such as *Ideonella sakaiensis* and *Thermobifida fusca* as well as from fungi such as *Fusarium solani*, *Humicola insolens*, and *Aspergillus oryzae* (Wang et al., 2008; Korpecka et al., 2010; Zimmermann and Billig, 2010). They are typically serine hydrolases and are characterized by a catalytic triad in their active site that consists of serine, histidine, and aspartate amino acids and an α/β hydrolase fold (Remington et al., 1992; Wei et al., 2014a). Most of the PET hydrolases that have been functionally verified also contain a C-terminal disulfide bond which is responsible for promoting thermal and kinetic stability (Roth et al., 2014; Sulaiman et al., 2014; Then et al., 2016). In general, carboxylic ester hydrolases are defined by their activity on *p*-nitrophenol (*p*-NP) acyl esters, whereby lipases act on medium to long-chain acyl esters (>C_10_), cutinases on short to medium-chain acyl esters (up to C_8_–C_10_), and esterases on short-chain acyl esters. Although PET hydrolytic activities are not correlated to *p*-NP acyl ester specificity, these substrates are useful for biochemical characterization of enzymes of interest (Kawai et al., 2020).

Polyethylene terephthalate hydrolytic enzymes are generating a good deal of interest from a biotechnological perspective, particularly with respect to their potential applications in areas such as biorecycling, biocatalysis, waste treatment and sustainable polymer modifications. Cutinases (EC 3.1.1.74) and cutinase-like enzymes, which are capable of processing high molecular weight polyesters, have become an important focus of PET hydrolase studies (Taniguchi et al., 2019). Numerous PET hydrolyzing enzymes have been biochemically characterized to date and are shown in (Table 1). Actinomycetes, especially *Thermobifida* species, are of interest for their PET hydrolytic cutinases and will be among the examples discussed. *I. sakaiensis*, which was discovered in PET-contaminated sediment that was sampled near a Japanese recycling facility, combines a cutinase-like hydrolase enzyme termed “PETase” with mono-(2-hydroxyethyl) terephthalate degrading “MHETase” to enable the use of PET as a carbon and energy source (Yoshida et al., 2016; Taniguchi et al., 2019). Strictly speaking, cutinases are defined by hydrolysis of cutin, but this is often left undetermined as cutin is not readily available commercially. Cutin degradation is not crucial for synthetic polyesterase activity, and PET enzymes are often categorized as cutinases, based on showing near-identical structures in crystallographic studies (Kawai et al., 2020). Cutinases that have been shown to hydrolyze cutin, and various polyesters, can do so under temperature conditions of 40–70°C and pH 7–9, without the help of cofactors (Furukawa et al., 2019).

**TABLE 1:** Native microbial PET hydrolytic enzymes that have been biochemically characterized and that are of known amino acid sequence.

Enzyme	Microbial source	NCBI accession number or PBD code	Seq length (aa)	Reaction temp (°C)	Substrate (crystallinity)	Reported degradation	References
**BsEstB**	*Bacillus subtilis* 4P3-11	ADH43200.1	489	40–45	3PET	TPA, MHET release	Ribitsch et al., 2011
**Cut190**	*Saccharomonospora viridis* AHK190	BAO42836.1	304	60–65	Amorphous PET film and package-grade PET	TPA, MHET release	Kawai et al., 2014
**FsC**	*Fusarium solani pisi*	1CEX	214	30–60	lcPET (7%) and bo-PET (35%)	5% lcPET weight loss	Griswold et al., 2003; Silva et al., 2005; Eberl et al., 2009; Ronkvist et al., 2009; Yoshida et al., 2016
**HiC**	*Humicola insolens*	4OYY	194	30–85	lcPET (7%) bo-PET (35%)	97 ± 3% lcPET weight loss	Ronkvist et al., 2009; Ribitsch et al., 2012a
***Is*PETase**	*Ideonella sakaiensis* 201-F6	GAP38373.1	290	20–45	lcPET (1.9%) and bottle-grade hcPET	TPA, MHET, EG release	Yoshida et al., 2016
**LCC**	Uncultured bacterium (from leaf-branch compost metagenome)	AEV21261.1	293	50–70	Amorphous PET film	≤5% weight loss	Sulaiman et al., 2012, 2014; Yoshida et al., 2016; Shirke et al., 2018
**PE-H**	*Pseudomonas aestusnigri*	6SBN	312	30	Amorphous PET film	MHET release	Bollinger et al., 2020
**PET12**	*Polyangium brachysporum*	A0A0G3BI90	298	50	PET nanoparticle agar	Zone of clearance	Danso et al., 2018
**PET2**	Uncultured bacterium (marine metagenome)	C3RYL0	308	50	PET nanoparticle agar	Zone of clearance	Danso et al., 2018
**PET5**	*Oleispira antarctica* RB-8	R4YKL9	310	50	PET nanoparticle agar	Zone of clearance	Danso et al., 2018
**PET6**	*Vibrio gazogenes*	UPI0003945E1F	298	50	PET nanoparticle agar	Zone of clearance	Danso et al., 2018
**Tcur0390**	*Thermomonospora curvata* DSM 43183	CDN67546.1	292	50	PET nanoparticle suspension	Reduced turbidity	Wei et al., 2014a
**Tcur1278**	*Thermomonospora curvata* DSM 43183	CDN67545.1	289	50–60	PET nanoparticle suspension	Reduced turbidity	Wei et al., 2014a
**TfCut1**	*Thermobifida fusca* KW3	CBY05529.1	319	55–65	lcPET film	≤11% weight loss	Then et al., 2015
**TfCut2**	*Thermobifida fusca* KW3	CBY05530.1	261	55–65	lcPET film	≤12% weight loss	Then et al., 2015
**TfH**	*Thermobifida fusca* DSM43793	WP_011291330.1	301	55	Bottle-grade PET (10%)	≈50% weight loss	Müller et al., 2005; Chen et al., 2008; Eberl et al., 2009; Silva et al., 2011
**Tha_Cut1**	*Thermobifida alba* DSM43185	ADV92525.1	262	50	3PET	TPA, HEB, MHET release	Ribitsch et al., 2012a
**Thc_Cut1**	*Thermobifida cellulosilytica* DSM44535	ADV92526.1	262	50	3PET and PET film (37%)	MHET, TPA, HEB release	Herrero Acero et al., 2011
**Thc_Cut2**	*Thermobifida cellulosilytica* DSM44535	ADV92527.1	262	50	3PET and PET film (37%)	MHET, TPA, HEB release	Herrero Acero et al., 2011
**Thf42_Cut1**	*Thermobifida fusca* DSM44342	ADV92528.1	262	50	3PET and PET film (37%)	MHET, TPA, HEB release	Herrero Acero et al., 2011
**Thh_Est**	*Thermobifida halotolerans* DSM44931	AFA45122.1	262	50	3PET	TPA, HEB, MHET release	Ribitsch et al., 2012b

Genomic and metagenomic strategies, which allow both genomic and gene expression analysis of unculturable and culturable microbes, have broadened the extent to which genetic information can be explored (Handelsman, 2004; Parages et al., 2016). The ever-increasing number of genomic and metagenomic datasets has led to the identification of genes of interest, including those encoding cutinases and PET hydrolase homologs (Hiraga et al., 2019). For example, functional screening of a leaf-branch compost metagenome enabled the isolation of LC-cutinase, a cutinase homolog with PET-degrading activity (Sulaiman et al., 2012). A metagenome mining approach that searches genome and metagenome databases has also recently been used to identify four novel PET hydrolase genes (Danso et al., 2018).

Genetic and protein engineering tools have commonly been used to increase the plastic degradation capacity of microorganisms and their enzymes, respectively (Wilkes and Aristilde, 2017; Jaiswal et al., 2020). For example, recombinant DNA techniques have enabled the expression of genes for pollutant degradation in a host that is better suited toward enzyme production, thus allowing purification of large quantities for application or further study. Substrate range and activity can be further enhanced using technologies such as site-directed mutagenesis. At the protein level, enzyme engineering has been employed to change or modify the protein’s amino acid sequence, which can lead to increased activity and tolerance to reaction conditions. The increased availability of enzyme structural information has greatly facilitated rational engineering approaches (Sharma B. et al., 2018; Jaiswal et al., 2020). For example, by introducing mutations, LC-cutinase, actinobacterial TfCut2, and *Is*PETase variant enzymes have been generated, amongst others, and have displayed improved PET hydrolyzing activities, owing to factors such as optimized catalytic properties and the relief of product inhibition (Wei et al., 2016; Kawai et al., 2020; Tournier et al., 2020).

In this review, microbial enzymes for PET hydrolysis will be examined, along with the current associated bottlenecks and considerations for progressing toward practical applications.

## Microbial Sources of PET Hydrolyzing Enzymes

### *Ideonella sakaiensis* Enzymes

A team of Japanese researchers, based in the Kyoto Institute of Technology and Keio University, have proposed three strategies that could be employed in the bioremediation and bio-recycling of PET waste together with other potential applications including bioconversions, microplastic, and microbead degradation, as well as PET-surface treatment (Taniguchi et al., 2019).

The first strategy is based on a microbial consortium named “no. 46”, which consists of bacteria, protozoa, and yeast-like cells, as revealed by light microscopy. This was isolated following extensive screening of environmental samples for PET-degrading microorganisms. The group screened wastewater, activated sludge, soil, and sediment from the site surrounding a PET bottle recycling plant (Taniguchi et al., 2019). The microbial consortium was found to both degrade PET and assimilate the degradation products into CO_2_ and water (Yoshida et al., 2016). No. 46 was also shown to adhere to PET film and create significant changes in its morphology, which could be visualized as film decay and/or surface whitening. PET film degradation occurred under ambient temperature conditions at a rate of 0.13 mg/cm^2^/day, with 75% of the carbon being catabolized (Yoshida et al., 2016). Furthermore, microbial consortium no. 46 was shown to retain PET degradation activity for at least 10 weeks and could be re-cultivated after freezing without losing activity. Of the estimated 20 types of bacteria within the consortium that have been identified, the following were investigated for their individual roles in the degradation process: *Bacillus megaterium*, which creates a biofilm on the PET film surface; *Rhizopus* sp., which acts within the biofilm to cleave the ester linkages of the PET polymer to give BHET; *Pseudomonas* sp., which further degrades BHET (bis-(2-hydroxyethyl)-terephthalate) into the monomers TPA and EG; and *Pigmentiphaga* sp. and *Mycobacterium* sp., which assimilate TPA and EG, respectively (Taniguchi et al., 2019).

*Ideonella sakaiensis* 201-F6, a bacterial strain which was later isolated from the consortium, provides the basis of the next degradation system (Tanasupawat et al., 2016). This bacterium is PET-lytic and its growth on minimal medium containing PET film has been shown to be much greater than on control medium without PET (Taniguchi et al., 2019). When grown in liquid culture, detection of PET hydrolysates was negligible, demonstrating the ability of *I. sakaiensis* to completely degrade and assimilate PET with CO_2_ as a complete oxidation product. The rate of *I. sakaiensis* degradation was around twice that of the microbial consortium no. 46 from which it was isolated. These *I. sakaiensis* bacterial cells were shown to adhere onto PET film via appendages that may also facilitate enzyme delivery during the attack (Taniguchi et al., 2019).

The final approach is based on employing novel enzymes identified in *I. sakaiensis*. A single open reading frame sharing 51% amino acid sequence identity with TfH, a known *T. fusca* PET hydrolase, was identified in the *I. sakaiensis* genome (Müller et al., 2005; Taniguchi et al., 2019). The corresponding recombinant protein released PET degradation products into aqueous medium and PET film exhibited crater-like pitting when exposed to the enzyme (Taniguchi et al., 2019). This *I. sakaiensis* cutinase-like enzyme, referred to as “PETase” (or *Is*PETase), was subsequently found to have the highest catalytic preference for PET, when compared with known PET hydrolytic enzymes such as TfH, *F. solani* fungal cutinase (FsC) (Silva et al., 2005) and LC-cutinase (LCC) (Sulaiman et al., 2012). These enzymes were assessed in terms of their activity against PET film and against highly crystallized, bottle-derived commercial PET (hc-PET), when incubated for 18 h at 30°C. When compared on this PET film, the activity of *Is*PETase was determined to be 120, 5.5, and 88 times as high as that of TfH, LCC and FsC, respectively (Yoshida et al., 2016). The efficiency and specificity of *Is*PETase with regards to PET hydrolysis makes it a promising candidate for new biodegradation strategies (Taniguchi et al., 2019). However, it is important to consider that *Is*PETase operates well under moderate temperature conditions, whereas the other enzymes included in this study are optimally active at higher temperatures due to their thermophilicity.

**FIGURE 2:**
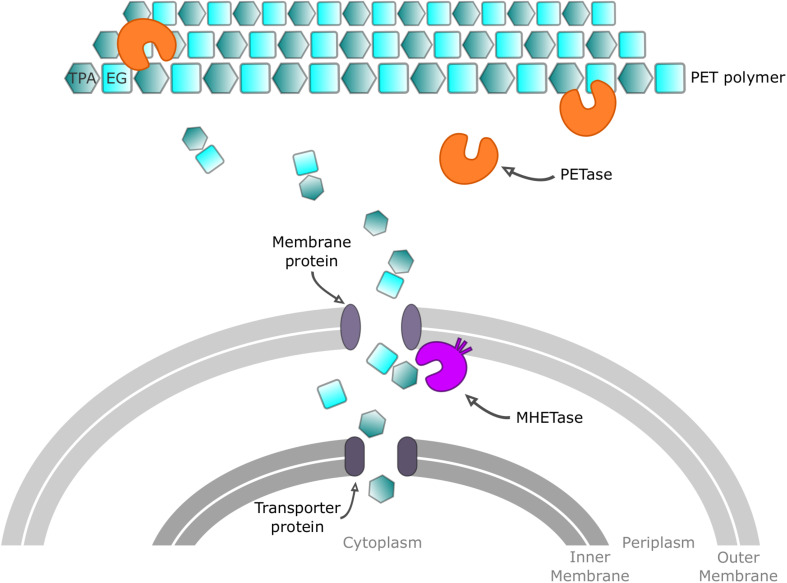
Proposed mechanism for PET processing in *Ideonella sakaiensis*. Extracellular PETase enzyme acts on PET polymer chains to give oligomeric mono-(2-hydroxyethyl) terephthalate (MHET), the monoester of terephthalic acid (TPA) and ethylene glycol (EG). Intermediates are transported through a membrane protein before reaching MHETase, which degrades MHET into TPA and EG. Finally, monomers are transported into the cytoplasm to be metabolized.

The primary product of *Is*PETase hydrolysis is mono(2-hydroxyethyl) terephthalate (MHET), which is broken down into the monomers, terephthalate (TPA) and ethylene glycol (EG), by a second *I. sakaiensis* enzyme called MHETase. This MHETase is a member of the tannase enzyme family and has been shown to degrade MHET into TPA and EG, with a catalytic efficiency (*k*_cat_/*K*_m_) of 4200 ± 370 s^–1^ mM^–1^. Interestingly, MHETase displays little activity on other ester compounds. Both PETase and MHETase enzymes were assigned new Enzyme Commission (EC) numbers, 3.1.1.101, and 3.1.1.102, respectively, and their role in a PET metabolic model was subsequently proposed (Figure 2). *Is*PETase, which acts extracellularly, first converts PET into oligomers (mainly MHET). PET hydrolysates are then transported, through an outer membrane protein (e.g., porin) into the periplasmic space and further hydrolyzed by MHETase into PET monomers (Taniguchi et al., 2019). MHETase is predicted to be a lipoprotein, which is likely to be anchored in the outer membrane. A lid domain, which confers its specificity and activity toward MHET, has also been proposed (Yoshida et al., 2016). A TPA transporter coupled with a TPA-binding protein is likely to be responsible for taking TPA up into the cytoplasm (Hosaka et al., 2013). It is thought that TPA subsequently enters the central tricarboxylic acid (TCA) cycle via protocatechuic acid, while EG is also catabolized in the TCA cycle via glyoxylic acid (Mückschel et al., 2012).

Structural analysis is important in providing useful information for subsequent protein engineering approaches, which may help in redesigning enzymes to make them more amenable for industrial applications (Taniguchi et al., 2019). Several groups have elucidated the crystal structure of *Is*PETase, revealing features shared by lipases (EC 3.1.1.3) and cutinases (EC 3.1.1.74), along with unique characteristics that differentiate the enzyme from cutinases previously recognized for PET hydrolysis (Han et al., 2017; Austin et al., 2018; Fecker et al., 2018; Joo et al., 2018; Liu et al., 2018). *Is*PETase has been expressed recombinantly from a codon-optimized gene in various *E. coli* host strains to facilitate generation of the crystal structure. The enzyme has a basic structure that resembles other PET hydrolyzing enzymes, featuring an α/β hydrolase fold and a nucleophile-His-acid catalytic triad. The α/β serine hydrolase family displays a catalytic mechanism based on a nucleophilic attack on the substrate’s ester bond by serine, which is activated via the other triad residues (Taniguchi et al., 2019). This involves serine linking to the ester carbonyl group to form a covalent tetrahedral intermediate that is later stabilized by two hydrogen bonds between amide bonds of the residues in a nearby oxyanion hole (Jaeger et al., 1994; Chen et al., 2013). In the case of *Is*PETase, the α/β hydrolase fold consists of nine β-strands that form a twisted central β-sheet conformation, surrounded by six α-helices (Chen et al., 2018), while Ser160-His237-Asp206 comprise the catalytic triad residues (Joo et al., 2018; Liu et al., 2019).

Upon structural comparison with known PET hydrolytic enzymes, including TfCut2 and LCC, *Is*PETase exhibits a notably wider active site and has an elongated substrate binding cleft, which is composed of two subsites (I and II). Four MHET moieties bind at subsites I, IIa, IIb, and IIc during PET degradation (Joo et al., 2018). Site I contains a cleavage site where ester bond breakage occurs. The enzyme also has an additional disulfide bond in its active site. The distinguishing features of *Is*PETase are detailed below (Taniguchi et al., 2019):

(1) In subsite II, a pair of Trp159-Ser238 residues ensure there is adequate space for the substrate to bind. PET binding is obstructed by residues at the corresponding site in cutinases (e.g., His169-Phe249 pair in TfCut2) (Taniguchi et al., 2019). The width of the *Is*PETase substrate binding pocket has been reported to be crucial for effective hydrolysis of crystallized PET (Liu et al., 2018).

(2) *Is*PETase has an extended connecting loop due to three additional residues, Ser245, Asn246, and Gln247. This also creates more space for PET to bind (Taniguchi et al., 2019).

(3) *Is*PETase has two disulfide bonds, 1 and 2 (Taniguchi et al., 2019). Previously studied cutinases only possess disulfide bond 1, with disulfide bond 2 being unique to *Is*PETase. This extra disulfide bond connects the alpha and beta loops which contain the catalytic triad. It is also responsible for higher active site flexibility compared to PET-active cutinases (Taniguchi et al., 2019). This flexibility enables *Is*PETase to accommodate the rigidity of the PET substrate without impairing the enzyme’s structural integrity. Removal of disulfide bond 2 results in reduced activity and weakening of the catalytic triad (Fecker et al., 2018).

Following a study based on structural and biochemical analyses of *Is*PETase, Joo et al. (2018) have proposed a detailed molecular mechanism for this enzyme. A four-MHET molecule called 2-HE(MHET)_4_ was used to mimic PET in a covalent docking model to facilitate elucidation of the substrate binding mode. At the catalytic center, triad residue Ser160 functions as a covalent nucleophile which acts on the carbonyl carbon atom of the ester bond, as seen in other carboxylesterase enzymes. The nitrogen atoms of amino acids Tyr87 and Met160 form an oxyanion hole which stabilizes the tetrahedral intermediate of the reaction. This stimulates the substrate binding site to form a long, shallow cleft, which is primarily hydrophobic. The first MHET moiety is bound to subsite I, while the second, third, and fourth moieties are accommodated by subsite II. Binding is mediated and stabilized largely by hydrophobic interactions, although certain residues also lead to polar and π–π interactions. Various site-directed mutagenesis experiments were used to verify the key residues in catalysis and substrate binding. This group also presented a molecular mechanism for the degradation of PET, which involves two steps: nick generation and terminal digestion. Once the secreted *Is*PETase has bound to the PET surface via its hydrophobic substrate binding cleft, the scissile ester bond is positioned closer to the nucleophilic Ser160. This enables the cleavage of one ester bond, creating a nick in PET and resulting in two PET chains with different terminals (TPA-terminal and HE-terminal), that are then digested into MHET monomers. As there are some variations in the digestion process depending on the terminal type, and because the digestions occur in a combinatorial manner, MHET, TPA, BHET, and EG accumulate, with BHET undergoing further degradation into MHET and EG. Interestingly, although *Is*PETase does not hydrolyze MHET to TPA and EG, a significant amount of TPA accumulates during the degradation reaction, which is believed to be related to digestion of the TPA-terminal described in this mechanism (Joo et al., 2018).

Furthermore, Chen et al. (2018) summarized recent crystallographic studies, in addition to their own work, to help uncover the key catalytic features of *Is*PETase and information regarding its underlying mechanism of action. Residue Trp156, which is found near the catalytic center, is thought to have a central role in binding the PET substrate. Although Trp156 is highly conserved across PET hydrolyzing enzymes, “Trp156 wobbling,” whereby the residue displays three different conformations, is exclusive to *Is*PETase. In this enzyme, “Trp156 wobbling” is coupled to Ser185, which corresponds to a bulky His residue in homologous structures, that would usually prevent Trp from adopting more than one conformation. A variant created by mutating Ser to His at position 185 resulted in decreased activity and indicated that the performance of *Is*PETase is at least partially influenced by the smaller Ser185 residue, as it allows more space for Trp156 to rotate (Han et al., 2017; Chen et al., 2018). Using substrate and product analogs of PET to study the substrate-binding mode, the group have also proposed a mechanism of action for *Is*PETase whereby the enzyme first forms a shallow, superficial cleft, within which “Trp156 wobbling” takes place. Next, primarily via hydrophobic interactions, PET substrate binds with its carbonyl group positioned at the catalytic center and its O atom facing the oxyanion hole, while the Trp156 indole ring interacts with the aromatic TPA moiety of PET. Then, successive formation of acyl-enzyme intermediate and cleavage of the ester bond occur in a nucleophilic attack by water (hydrolytic reaction). Finally, the product is rotated and released from the catalytic center following interaction of the benzoic acid group with Trp156 (Chen et al., 2018).

With respect to PET hydrolase enzymatic mechanisms, while much progress has been made, the exact details remain to be conclusively determined. In response to Joo et al. (2018), another group have argued that the flexible oligomeric 2-HE(MHET)_4_ substrate employed in this study is not representative of the highly rigid PET polymer chain, and it is unlikely that the *Is*PETase catalytic mechanism involves simultaneous binding of all four MHET moieties and the occurrence of conformational changes in PET that perfectly match the binding cleft (Wei et al., 2019). Instead, Wei et al. (2019) propose that hydrolysis of amorphous PET is facilitated by weak interactions between its aromatic phenylene units and the nearby hydrophobic amino acids of the enzyme. This hypothesis is based on phenylene ring motions, which are more inclined to arise compared with the rigid EG units that directly flank the ester bond (Wei et al., 2019). Although cutinase catalytic mechanisms have also been explored by studying enzyme structures complexed with inhibitors and natural substrate analogs, the precise binding mode of PET and the underlying mechanism of cutinase PET hydrolysis requires further investigation, as the structure of PET is very different to cutinase substrates, such as cutin and triglyceride (Han et al., 2017).

While *I. sakaiensis* and its enzymes are well-studied and have gained much interest as a PET degradation system, other relevant microorganisms and enzymes have also been identified and will now be discussed.

### Actinobacterial Enzymes

*Thermobifida fusca*, a thermophilic actinomycete, is one of the multiple Actinobacterial strains that have been recognized as producers of enzymes for PET hydrolysis (Müller et al., 2005; Wei and Zimmermann, 2017a). *T. fusca* DSM43793 has been shown to produce two similar hydrolases, BTA-1 (commonly referred to as TfH) and a BTA-2, while *T. fusca* KW3, a moderate thermophile, has been reported to produce TfCut1 and TfCut2 cutinases (EC 3.1.1.74). *T. fusca* YX has been shown to produce Tfu_0882 and Tfu_0883, which are lipases (EC 3.1.1.3) (Wei and Zimmermann, 2017a). In one study, Thf42_Cut1 cutinase from *T. fusca* DSM44342 was reported to hydrolyze PET alongside Thc_Cut1 and Thc_Cut2 cutinases from another *Thermobifida* species, namely *Thermobifida cellulosilytica* (Herrero Acero et al., 2011). Additionally, *Thermobifida alba* and *Thermobifida halotolerans* are of interest for their PET hydrolyzing enzymes (Ribitsch et al., 2012a, b). Other known actinobacterial PET hydrolytic enzymes that have been described include those from the genera *Saccharomonospora* and *Thermomonospora* (Table 1).

TfH (BTA-1), a cutinase enzyme which was isolated by Müller et al. (2005), was used to degrade melt-pressed PET bottles over a 3 week period at 55°C, with its weight being reduced by approximately 50%. This offered a marked improvement over the chemical hydrolysis of PET, which typically requires much higher temperatures, and had the added benefit of reducing unwanted side products and generating monomers of higher purity for repolymerization. While TfH shares 65% sequence similarity with a lipase from *Streptomyces albus*, it differs from lipases, which can only break ester bonds at a hydrophobic surface, in that it has additional esterase activity that enables hydrolysis of dissolved esters (Mueller, 2006).

*Thermobifida fusca* hydrolases BTA-2, Tfu_0882, TfCut1, TfCut2, and TfH have been assessed for their degradation capabilities on amorphous (non-crystalline) PET film (Wei and Zimmermann, 2017a). When incubated between 55 and 65°C for 48 h, film weight losses of up to 4, 5, 11, 12, and 14% were observed for these five enzymes, respectively (Then et al., 2015; Wei and Zimmermann, 2017a). TfCut2 variants have been shown to achieve 25% weight loss of the same film using temperature conditions of 65–80° for 48 h (Then et al., 2015) and 45% after 50 h incubation at 65°C (Wei et al., 2016). Both TfCut1 and TfCut2, along with their variants, have already been successfully applied in textile finishing processing for PET fiber surface modification (Zimmermann and Billig, 2010; Wei et al., 2014b).

In a study on TfCut2 cutinase, the enzyme was crystallized in both free and inactivated forms before structural and functional characterization (Roth et al., 2014). Combined with molecular models, the structural components responsible for substrate and product specificity were analyzed. TfCut2 displayed a standard α/β hydrolase fold and a S130-H208-D176 catalytic triad was found in a crevice on the enzyme surface and the highly resolved structure provided an appropriate foundation for enzyme engineering. The thermostability of TfCut2 was also investigated by comparison with a less stable *T. alba* AHK119 homolog. While the melting temperature (*T*_m_) of TfCut2 appeared to be 70°C when measured by temperature-dependent CD spectroscopy, loss of activity was measured experimentally at 61°C (Roth et al., 2014). Despite this, TfCut2 displayed improved thermal stability when compared with other characterized bacterial cutinases (EC 3.1.1.74). It is believed that denaturation may be prevented in TfCut2 by the presence of a disulfide bridge between Cys241 and Cys259 which may stabilize a highly flexible region nearby that surrounds residues 245–247. Residues at the boundary of the active site also exhibit high levels of flexibility in relation to their overall structure, possibly helping to enable induced fit for catalysis. Flexibility in some areas can be susceptible to local unfolding which might explain the lower *T*_m_ seen in the activity assay. This analysis also suggested that the optimized amino acid sequence pattern and hydrogen bond network of this enzyme contribute to its thermostability (Roth et al., 2014; Taniguchi et al., 2019).

Thc_Cut1, Thc_Cut2, and Thf42_Cut1, three cutinases which are closely related, were compared based on their hydrolytic activities toward bis(benzoyloxyethyl)-terephthalate (3PET), a short-chain PET model substrate, in addition to PET film with 37% crystallinity (Herrero Acero et al., 2011). The enzymes were incubated at 50°C with the 3PET assay proceeding for 72 h and the PET film treatment requiring 120 h, shaking at 130rpm. HPLC (high-performance liquid chromatography) was used to quantify the resulting hydrolysates. Of the three enzymes tested, Thc_Cut1 was found to release MHET and TPA from both PET and 3PET to the greatest extent. For both Thc_Cut1 and Thf42_Cut1, TPA was found to be the major product of hydrolysis, indicating more rapid processing of MHET, which was in higher abundance following treatment using Thc_Cut2. Although Thc_Cut1 and 2 had 93% homology, varying enzyme surface properties appeared to be responsible for the significant activity differences observed (Herrero Acero et al., 2011).

Another cutinase enzyme from *T. alba* called Tha_Cut1 was assessed for hydrolysis of 3PET and PET films (Ribitsch et al., 2012a), based on the previously mentioned methods used for Thc_Cut1 and 2 (Herrero Acero et al., 2011). For the 3PET assay, the well-studied fungal cutinase HiC was compared with Tha_Cut1 and although HiC released twice as many overall hydrolysates, it was determined than the two enzymes had distinct substrate specificities, with Tha_Cut1 releasing 35 times the amount of a hydrolysis product called 2-hydroxyethyl benzoate (HEB). This comparison provided further insights into the mechanism of Tha_Cut1, revealing the main products of 3PET hydrolysis to be MHET and HEB, indicating efficient hydrolysis of the ester bond between them. Tha_Cut1 displayed a high similarity to Thc_Cut1 from *T. cellulosilytica*, with a difference of just four amino acids that are located in a key region involved in substrate-enzyme interaction (Ribitsch et al., 2012a). In addition, two cutinase-like polyesterase enzymes Est1 and Est119 from tandem genes in *T. alba* AHK119 have been shown to have broad substrate specificity for various polyesters. Purified Est119 has been biochemically characterized, revealing highest kinetic activities (*V*_max_ and *k*_cat_) toward short-chain *p*-nitrophenyl butyrate (*p*-NPB). However, the enzyme *K*_m_ values showed a higher affinity for longer chain (C_6_ and C_8_) acyl esters, indicating that Est119 is a cutinase-like enzyme rather than a true lipase or esterase (Thumarat et al., 2012). For both Est119 and Est1, optimum temperature and pH were recorded as 50°C and pH 6.0, respectively (Thumarat et al., 2015). Despite the enzymes displaying 98% similarity with each other, Est1 displayed higher activity and thermostability (Kawai et al., 2013). More recently, an Est1 (A68V/T253P) variant was shown to have the highest thermostability when compared with wild-type and mutant Est1 and Est119 enzymes. Est1 (A68V/T253P) was assayed with 3PET and PET film, shaking for 3 h at 50°C, and was noted for its PET surface hydrophilization and polyester degradation abilities (Thumarat et al., 2015).

Cut190 is a cutinase enzyme from *Saccharomonospora viridis* AHK190. Following mutational analysis, one variant Cut190 (S226P/R228S) that was generated, displayed particularly high activity and thermostability (Kawai et al., 2014). This variant remained stable up to 65°C and over a pH range of between 5 and 9 when incubated for 24 h, while also retaining 40% of its activity when incubated at 70°C for 1 h. Various aliphatic and aliphatic-co-aromatic polyester films were also shown to be efficiently degraded by Cut190 (S226P/R228S) and the enzyme even hydrolyzed PET film above 60°C. Both the wild-type and mutant Cut190 are dependent on Ca^2+^ ions to assist in their activity and thermostability (Kawai et al., 2014).

Tcur0390 and Tcur1278 are two hydrolase enzymes from *Thermomonospora curvata* DSM43183, the genes for which were identified following genome scanning analysis of the strain and were found to display 61% sequence identity with TfCut2 from *T. fusca* (Wei et al., 2014a). Enzyme activity was subsequently investigated using polycaprolactone (PCL) and polyethylene terephthalate (PET) nanoparticle assays. While Tcur0390 displayed higher hydrolytic activity than Tcur1278 at temperatures up to 50°C, only Tcur1278 was capable of hydrolyzing PET nanoparticles when temperatures were elevated to 55 and 65°C. Relatively low thermal stability was observed for both Tcur1278 and Tcur0390 at their optimal temperatures (60 and 55°C, respectively), with an irreversible activity loss of over 65% following incubation for just 10 min. The optimal pH for both enzymes is pH 8.5 (Wei et al., 2014a).

In a study that employed an *in silico*-based screening approach to interrogate 52 genomes from the *Streptomyces* genus, a potential PETase-like gene was identified in *Streptomyces* sp. SM14 (Almeida et al., 2019). Heterologous expression of the gene in *Escherichia coli* resulted in the extracellular production of an enzyme, SM14est, that was shown to have polyesterase activity on polycaprolactone, the model substrate for plastic degradation (Almeida et al., 2019). SM14 is a marine sponge-derived isolate which may have been exposed to plastics and/or microplastics, resulting from its association with the filter-feeding sponge *Haliclona simulans*. Subsequent amino acid sequence analysis and comparison of SM14est with the well-described *Is*PETase revealed that the serine hydrolase motif Gly-x1-Ser-x2-Gly and the Ser-Asp-His catalytic triad are conserved in both enzymes. However, SM14 displays key differences in its catalytic sub-site II residues as well as an absence of the two disulfide bonds and the extended loop featured in *Is*PETase, which may contribute to a reduced efficiency in PET binding and hydrolysis (Joo et al., 2018; Almeida et al., 2019).

Bacterial enzymes of interest for PET hydrolysis are not limited to those derived from actinomycetes, with other examples including a *Bacillus subtilis* esterase (Ribitsch et al., 2011), a cutinase from *Pseudomonas mendocina* (Yoon et al., 2002), and a *Burkholderia* spp. lipase (Lee and Chung, 2009). In 2019, the draft genome sequences of five bacterial isolates, from *Bacillus* and *Pseudomonas* species, were deposited in Genbank (León-Zayas et al., 2019). As a consortium, these isolates synergistically degraded 100mg of granular PET to give a weight loss of 3.15 mg (∼3% reduction rate), following 6-weeks at 30°C (León-Zayas et al., 2019). Most recently, Bollinger et al. (2020) identified a polyester degrading carboxylesterase (EC 3.1.1.1) within the genome of the mesophilic marine bacterium *Pseudomonas aestusnigri*. The enzyme, named PE-H, was revealed as a PET hydrolytic enzyme (type IIa), based on amino acid sequence homology. The group also solved the enzyme’s crystal structure, which featured the canonical α/β hydrolase fold and showed high homology to known polyesterases. PE-H was shown to hydrolyze amorphous PET film at 30°C, with intermediate MHET released as a product. Although the wild-type enzyme failed to hydrolyze a commercial PET bottle-based film, a variant of PE-H (Y250S), which displayed some hydrolytic activity toward PET in this form, was obtained using rational mutagenesis (Bollinger et al., 2020).

### Fungal Enzymes

Cutinases (EC 3.1.1.74) from fungi have also shown activity toward PET as a substrate, with members of the *Fusarium* and *Humicola* genera, representing the most significant sources of these enzymes (Danso et al., 2019).

The activities of *Fusarium solani pisi* cutinase (FsC) and *Humicola insolens* cutinase (HiC) have previously been evaluated, on both low-crystallinity and biaxially oriented PET films having 7 and 35% crystallinity, respectively (Ronkvist et al., 2009). Using a pH-stat based assay, the cutinases were shown to be 10-fold more active on low crystallinity PET over the biaxially oriented film. HiC was shown to be capable of completely degrading low crystallinity PET film with 97% (±3%) weight loss being observed within 96 h. Further analysis of degradation products indicated that the water-soluble degradation products consisted exclusively of TPA and EG. Despite its preference for amorphous PET, the HiC enzyme was sufficiently active on crystalline PET regions to enable its full degradation. In comparison, a weight loss of just 5% was achieved by the FsC enzyme on low-crystallinity PET film. While FsC performed optimally at 50°C, HiC displayed higher thermostability, maintaining maximum initial activity between 70 and 80°C (Ronkvist et al., 2009).

More recently, HiC has been shown to act synergistically with *Candida antarctica* lipase B (CALB) to effectively hydrolyze PET to TPA (Carniel et al., 2017). Initial screening of 10 lipases using the PET intermediate bis-(2-hydroxyethyl)-terephthalate (BHET), revealed that CALB could complete its conversion to TPA, while HiC was limited by the last reaction step. When evaluated for PET hydrolysis, HiC displayed significant accumulation of the intermediate MHET, despite showing better potential for depolymerization of PET. By combining the CALB enzyme with HiC, PET degradation was shown to be more complete, resulting in a 7.7-fold increase in TPA yield from PET (Carniel et al., 2017).

*Aspergillus oryzae*, *C. antarctica*, and *Penicillium citrinum* are among other fungal enzymes that have been investigated for activity on PET and are considered most suitable for PET fiber and fabric treatments (Zimmermann and Billig, 2010; Kawai et al., 2019).

### Metagenome-Derived Enzymes

Novel genes from the unculturable fraction of different environmental ecosystems can be accessed using metagenomic approaches. During a metagenomic study of leaf-branch compost, a gene with 57.4% sequence identity to Tfu_0883 cutinase from *T. fusca* was identified (Sulaiman et al., 2012). This gene was found to encode LC-cutinase (LCC), a novel cutinase homolog, following a functional-based screen of a metagenomic library from the compost using tributyrin agar. LCC was subsequently heterologously expressed in *E. coli* and shown to hydrolyze various fatty acid monoesters optimally at 50°C, with a preference for short-chain substrates (up to C_4_). This reaction was used to show that the optimal enzymatic activity for LC-cutinase occurs at 50°C and pH 8.5. The enzyme’s specific activity for *p*-NPB is comparable to that of the *T. fusca* cutinase, although slightly lower. LCC was also capable of degrading PCL and PET, with the enzyme displaying specific PET-degrading activity of 12 mg/h/mg of enzyme at 50°C and pH 8.0 (Sulaiman et al., 2012).

A dual enzyme system, which combined LC-cutinase with an immobilized *T. fusca* KW3 carboxylesterase (EC 3.1.1.1) called TfCa, was subsequently investigated for its ability to hydrolyze PET films (Barth et al., 2016). Typically, enzymatic PET degradation is restricted once the MHET intermediate is formed. The presence of immobilized TfCa was, however, shown to enable LC-cutinase hydrolysis of inhibitory MHET. HPLC analysis of degradation products indicated that the LCC-TfCa dual enzyme system gave a 2.4-fold higher yield of hydrolysates compared with one that combined TfCa with TfCut2 from *T. fusca*. In terms of total amounts of products released with the LCC-TfCa combination, an increase of 104% was observed versus PET enzymatic hydrolysis where TfCa was not added (Barth et al., 2016).

A recent study by the Streit group, involving a search for PET hydrolase genes across existing marine and terrestrial metagenomic datasets uncovered over 800 putative PET hydrolases (Danso et al., 2018, 2019). 13 potential PET homologs (PET1-PET13) were selected based on their sequence similarity to previously identified PET hydrolase enzymes. Of these, four enzymes, PET2, PET5, PET6, and PET12, were functionally verified as novel PET hydrolase candidates, with active clones giving clearance on PET nanoparticle plates. One of these enzymes, PET2, which was obtained from marine metagenomic data, was shown to have a temperature optimum of 70°C and retained its thermostability up to 90°C (Danso et al., 2018).

Screening of over 200 purified, uncharacterized hydrolytic enzymes from environmental metagenomes and sequenced microbial genomes was carried out in a recent study to find proteins with strong degradative activity against synthetic polyesters (Hajighasemi et al., 2018). The selected hydrolase genes were heterologously expressed in an *E. coli* host and purified by a combination of affinity chromatography and either ion exchange or size exclusion chromatography. Among the enzymes identified were MGS0156 and GEN0105, two metagenomic esterases capable of hydrolyzing polylactic acid (PLA), polycaprolactone and a PET model substrate, 3PET. Following determination of its crystal structure, MGS0156 was found to feature a modified α/β hydrolase fold, a highly hydrophobic active site, and a lid domain. Structure-based mutational studies revealed the key amino acid residues required for hydrolytic activity, including catalytic triad residues Ser232, His73, and Asp350, and adjacent residues His231, Lys233, and Asp372. The side chains of Cys173 and Cys287 formed a disulfide bond which stabilizes an important protein lid domain. The polyesterase activity of a MGS0156 mutant (L169A) was twice as high as that of the wild-type (Hajighasemi et al., 2018).

Metagenomic tools have a powerful ability to tap into the microbial biodiversity of different environments. Thus, it is worth further developing search algorithms that explore metagenomic datasets for valuable activities as well as establishing consistent functional assays that can be run in parallel to detect PET hydrolase enzymes (Danso et al., 2019).

## Challenges and Limitations

While the area of enzymatic PET hydrolysis is expanding rapidly, several major challenges remain which will need to be addressed to further advance the field.

### Low Prevalence of PET Hydrolysis Activities

So far, relatively few microbial genera have been described which possess the ability to degrade PET, with those that do often only causing partial break down into oligomers (Wei and Zimmermann, 2017b). Current reports indicate that the PET degradation trait features in only a limited number of bacterial phyla, with most to date being found in members of the Actinobacteria (Herrero Acero et al., 2011).

**FIGURE 3:**
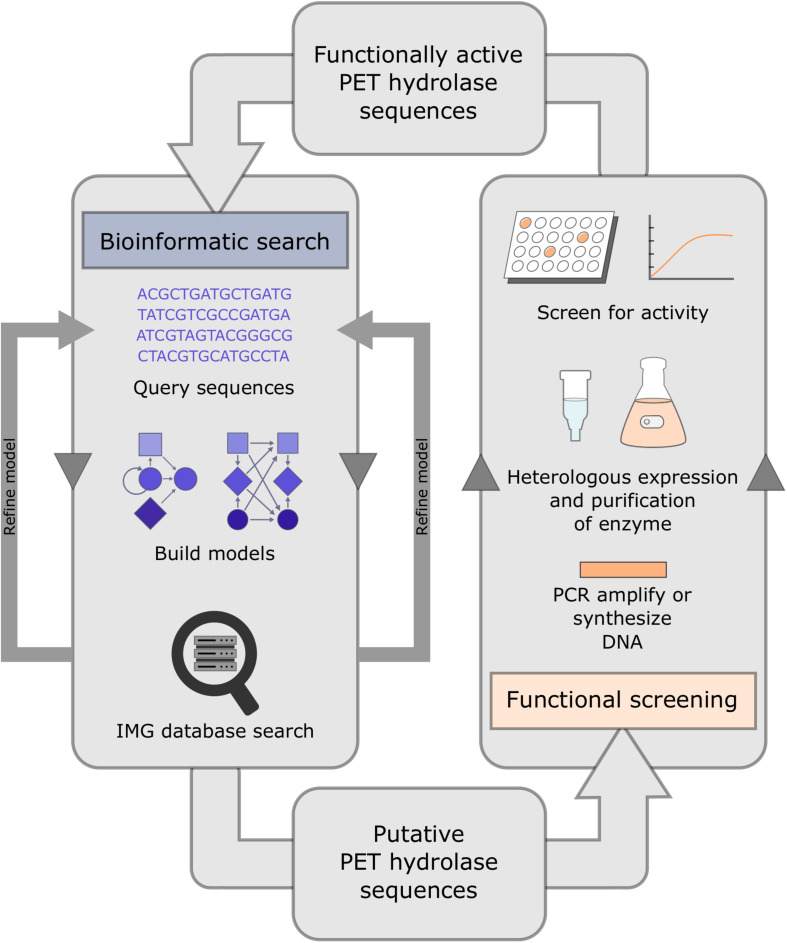
System for the identification and functional testing of candidate PET hydrolase enzymes. Potential PET hydrolase sequences may be uncovered with an initial bioinformatic search of existing databases, by employing hidden Markov models. Candidate genes are then cloned into a heterologous host to verify activity against substrates, e.g., PCL and PET-nanoparticles.

In the aforementioned study targeting novel genes related to PET hydrolysis in marine and terrestrial metagenomics datasets, Danso et al. (2018) employed a search algorithm that detected 504 possible candidate genes from various databases. In addition, they performed a global search of over 16 Gb of metagenomic sequence data from the Integrated Microbial Genome (IMG) database, which resulted in the identification of 349 putative PET hydrolases. Both searches relied on a hidden Markov model and were followed by functional testing of potential PET hydrolase sequences (Figure 3). PET hydrolase frequencies ranged from 0.004 to 0.92 hits/Mb and 0.0001 to 1.513 hits/Mb for marine and terrestrial metagenome datasets, respectively. An average of 157 PET hydrolase homologs were present in terrestrial metagenomes, compared with an average of 42 homologs in marine metagenomes. A metagenomic sample from a crude oil reservoir offered the highest rate of sequence hits (1.5 hits/Mb). While the phylum Actinobacteria was the main provider of terrestrial-derived enzymes, many PET hydrolases predicted from the marine samples were linked to Bacteroidetes (Danso et al., 2018).

Bacteroidetes species, although not previously associated with PET hydrolysis, are known to be equipped with multiple hydrolases and binding molecules for degradation of other polymers such as xylan, cellulose, and pectin (Dodd et al., 2011; Thomas et al., 2011). The second most abundant phylum associated with both datasets was the Proteobacteria, comprising 20 and 30% of hits in the terrestrial and marine data, respectively. Beta-, Delta-, and Gammaproteobacteria were the main hosts harboring PET hydrolase genes within this phylum. Since over 100 metagenomes were included in the analysis, an overall picture of the occurrence of PET hydrolase enzymes was generated. However, this data may not be an accurate representation of the expression of these genes in the natural environment. The low gene frequencies observed suggests that this trait may have only evolved quite recently and may not have had the opportunity to spread within different microbial populations. Overall, the data suggests that PET hydrolases are rare enzymes (Danso et al., 2018). Notwithstanding this, it is also likely that by exploiting global metagenomic data sets to gain access to dark matter proteins and non-culturable microbes, that there is potential to increase the diversity of microbes and enzymes that act on synthetic polymers (Danso et al., 2019).

### Impact of PET Properties on Enzymatic Activity

The success of PET hydrolysis relies on a balanced combination of suitable enzyme structure and polymer chain flexibility (Zumstein et al., 2017). There are a few factors, relating to the nature of PET, which influence the extent to which it is hydrolyzed. PET crystallinity and orientation are among the key features which may affect the ability of an enzyme to break apart its building blocks (Webb et al., 2013; Kawai et al., 2019).

**FIGURE 4:**
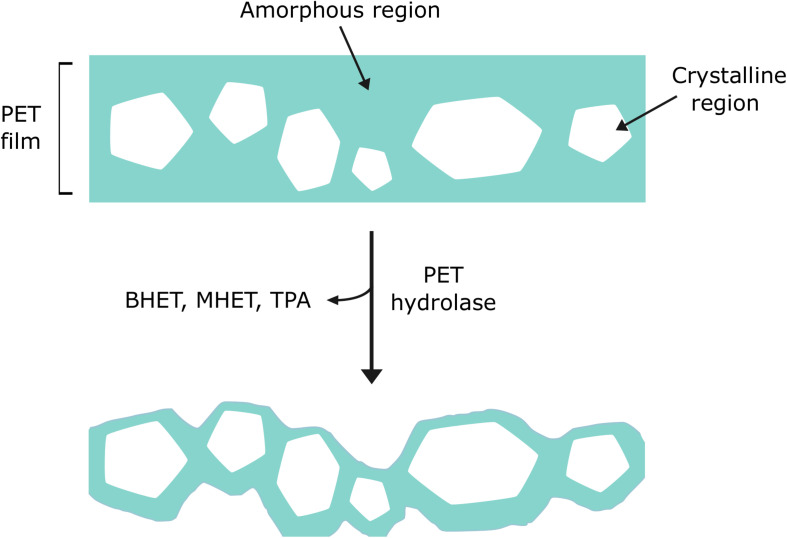
Enzyme preference for amorphous regions of PET over crystalline regions. PET hydrolase enzymes have been shown to favor lower crystallinity, amorphous areas of PET (shown in blue).

As with most polymers, PET is typically comprised of a complex structure with crystalline regions that feature tightly packed chains in parallel, and amorphous regions where the chains are disordered (Demirel et al., 2011; Robertson, 2016). Depending on the intended use of the final product, PET can have different degrees of crystallinity. Generally, PET employed to make bottles and textiles is associated with having a high crystallinity of between 30 and 40%. Conversely, PET which is used to create packaging has lower crystallinities, for example, PET-GF, a commercially available low-crystallinity PET film, is estimated to have 6–7% crystallinity (Kawai et al., 2014). Numerous reports indicate that PET hydrolase enzymes preferentially degrade the regions of PET that are amorphous in nature (Figure 4) (Welzel et al., 2002; Brueckner et al., 2008; Ronkvist et al., 2009; Donelli et al., 2010; Gamerith et al., 2017). As the crystallinity of PET increases, the flexibility and movement of the polymer chains becomes more restricted, thus reducing the susceptibility of these chains to enzymatic attack. In spite of this, it has been suggested that hydrolysis of amorphous regions may impact on crystalline regions, such that both can ultimately be digested (Ronkvist et al., 2009; Kawai et al., 2019). Furthermore, the repeating units of bulky aromatic terephthalate, which are present in the PET backbone, restrict polymer chain mobility and therefore, degradability (Wei and Zimmermann, 2017b).

To achieve the desired form for bottles and textiles, the degree of orientation is increased by stretching to order the PET polymer chains (Kawai et al., 2019). PET can also be biaxially oriented (*bo*-PET) whereby the plastic undergoes additional stretching in two directions (Breil, 2013). In both cases, orientation results in strengthening of the hydrogen bonds between the polymers and further induces crystallization (Wei and Zimmermann, 2017a). In addition, crystallinity and orientation during production will influence surface topology, as seen in the manufacture of plastic bottles by injection molding – different parts of the bottle will have different surface properties with varying orientations of polymer chains. While these methods enhance the physical properties of PET for application, it is very restrictive in terms of enzymatic degradation and there are currently no PET hydrolases known to directly digest PET in any of the forms just mentioned (Kawai et al., 2019).

The temperature range within which a polymeric substrate changes from a glass-like and rigid state to being soft but not yet melted, is referred to as the glass transition temperature (*T*_g_) (Becker and Locascio, 2002). The application of heat above *T*_g_ is typically used to induce crystallization, normally accompanied by molecular orientation. High crystallinity (and therefore increased resistance to degradation) is associated with higher glass transition temperatures, with amorphous PET having a *T*_g_ of 67°C and crystalline PET having a *T*_g_ of 81°C (Groeninckx et al., 1974; Demirel et al., 2011). However, when PET is in aqueous conditions, *T*_g_ values are lowered to 60–65°C as a result of the diffusion of water molecules between the polymer chains (Kikkawa et al., 2004; Kawai et al., 2014). This weakens the associated hydrogen bonds and thereby increases randomness, flexibility, and mobility of the chains. Enzymatic reactions are routinely performed in a solution, meaning that the *T*_g_ of PET decreases such that enzymes have increased accessibility to the polymer chains. Water absorbency of PET can vary between 0.1 and 1% depending on the form but will also increase under higher temperatures. Therefore, it is recommended to set high PET hydrolysis reaction temperatures, preferably above *T*_g_, as this has been reported to give higher rates of degradation (Kawai et al., 2014, 2019; Oda et al., 2018). On the other hand, it is then vital to employ a thermostable PET hydrolase. Thermostability is generally advantageous as catalysis is promoted at raised temperatures and allows biocatalysis to be maintained over a long period at ambient temperatures (Kawai et al., 2019).

Inhibition of the cutinase enzyme TfCut2 by the intermediate products of PET degradation has been investigated in a recent study aiming to identify the bottlenecks in reaching complete PET hydrolysis (Barth et al., 2015). MHET, BHET, EG, and TPA are recognized as the main hydrolysis products of PET films and fibers (Vertommen et al., 2005; Brueckner et al., 2008; Eberl et al., 2009). The experiment involved TfCut2 digestion of a PET nanoparticle substrate in the presence of these four intermediates, with their inhibitory effect analyzed using reversed-phase HPLC and a model for heterogenous enzymatic polymer hydrolysis. MHET and BHET were shown to competitively inhibit hydrolysis by occupying the substrate binding site of the TfCut2 enzyme whereas TPA and EG had no impact on the ability of the enzyme to degrade the PET nanoparticles (Barth et al., 2015). One way of overcoming this challenge has been to use other enzymes in combination with PET hydrolases to improve substrate binding and catalytic characteristics (Wei et al., 2016; Carniel et al., 2017; Austin et al., 2018).

When studying and comparing PET hydrolyzing enzymes, it is important to account for the variability in properties of different PET substrates. While commercial PET-GF film is regularly used and is a suitable standard for comparing the abilities of PET hydrolases, it has not been employed across all studies. It is also necessary to consider the chosen method for detecting and reporting activity, i.e., weight loss, hydrolysis, etc. In addition, many enzymes, including HiC, TfH, FsC, and Cut190 have been used in the textiles industry to achieve more desirable PET fiber characteristics and this requires only surface modification and avoids degrading the inner PET building block. While PET hydrolases may be used for surface treatments, they are distinct from PET surface-modifying enzymes. PET hydrolases should significantly degrade the building blocks of PET (by 10% at least) and result in visible change when observed by scanning electron microscope (SEM) (Kawai et al., 2019).

More recently, this group further discussed the need for clarification when categorizing an enzyme as a PET hydrolase (Kawai et al., 2020). They have recommended grouping PET hydrolyzing enzymes into, (1) PET hydrolases and (2) PET surface-modifying enzymes. They suggest that the only enzymes which can currently be considered as PET hydrolases are the following cutinases; HiC, LCC and variants of TfCut2 (Wei et al., 2016) and Cut190 (Oda et al., 2018). This is based on criteria for efficient PET degradation, namely thermostability (>65°C, or ideally >75°C) and the capacity for the open active site to bind multiple MHET monomers. It follows that mesophilic *Is*PETase, despite its structural similarities to these cutinases and observed hydrolyzing activities at 30°C, should not be regarded as a PET hydrolase, particularly with respect to application (Kawai et al., 2020). It was also recently demonstrated that while amorphous PET supports growth of *I. sakaiensis*, high crystallinity PET films and bottles do not (unless hcPET is melted and cooled rapidly to give amorphous plastic) (Wallace et al., 2020). TfH may still be grouped with PET hydrolases as it was the first enzyme reported for efficient PET hydrolysis and it is thermophilic.

It is also worth noting that solubilizers and other additives, which are often mixed into plastics to change their properties, may interfere with the activity of degradative enzymes and may also account for some weight loss during enzymatic digestion due to microbial break down of these chemical agents (Danso et al., 2019). While this does not usually apply to PET manufacturing, plasticizers may be used for some PET products (Hahladakis et al., 2018). A simple and robust PET powder-based suspension assay was recently used to quantify product release from PET, but product detection was shown to depend on choice of analytical technique. Therefore, it is advisable to use a combined approach. In this study, a plate reader method (for rapid screening, measurement of initial rates, and comparative biochemical studies) was well complemented by reversed-phase HPLC analysis (for more in-depth information on the aromatic monomers produced), which offered greater insight and avoided errors (Bååth et al., 2020).

Although these factors can create difficulty in making exact conclusions regarding the ability of an enzyme to hydrolyze PET, this is a relatively new field of research which should see greater harmony across different studies as it progresses into the future. In fact, the recommendations provided by Kawai et al. (2020), for distinguishing PET hydrolases from other enzymes may be worthy of further consideration in this area, particularly for comparative purposes when assessing the potential of new PET-active enzymes and when considering PET hydrolases for practical use.

### Enzyme Properties, Catalytic Efficiency, and Thermal Stability

So far, the consensus in the area of PET hydrolysis has been that known PET hydrolase enzymes have low turnover rates, particularly toward highly crystallized forms of PET (Wei and Zimmermann, 2017a; Liu et al., 2018; Danso et al., 2019; Taniguchi et al., 2019). Even *Is*PETase, which has shown specificity for degradation of hc-PET, is considered to have a relatively low catalytic activity (Taniguchi et al., 2019). Furthermore, given that reaction temperatures above the *T*_g_ of PET have been advised for more rapid hydrolysis, candidate enzymes should ideally display high enzymatic efficiency while also retaining activity over 75°C (Yoshida et al., 2016; Taniguchi et al., 2019). For instance, *Is*PETase is heat labile but thermostable cutinases such as LCC, TfH, and HiC may fall short of *Is*PETase in terms of specificity for PET substrate, demonstrating the need for a combination of both traits (Wei and Zimmermann, 2017a; Liu et al., 2018; Taniguchi et al., 2019). Enzyme adsorption, whereby the protein adheres to the polymer surface, is a key process in the hydrolysis of PET and other polymers. This binding results from contact between hydrophobic PET fibers and any hydrophobic amino acid residues located at the protein surface (Norde, 1996; Kim and Yoon, 2002; Stuart, 2003; Palonen et al., 2004; O’Neill et al., 2007). It was mentioned already that *T. cellulosilytica* cutinase, Thc_Cut2, displayed lower hydrolytic efficiency compared with Thc_Cut1 that could be attributed to the individual hydrophobic surface properties of these enzymes. Subsequently, site-directed mutagenesis was used to demonstrate that amino acids on the enzyme surface are important for PET hydrolysis, by exchanging selected Thc_Cut2 residues with those only present on the surface of Thc_Cut1 (Herrero Acero et al., 2013). Another group improved Thc_Cut1 PET hydrolysis through fusion of two hydrophobic binding domains to enhance the enzyme’s adsorption abilities (Ribitsch et al., 2013). Also, microbial biofilm production can play an important role in plastic biodegradation due to the comparably high hydrophobicity of the associated cell surfaces, which in turn facilitates their attachment to synthetic polymers. Given that cell surfaces are usually hydrophilic in nature, attachment is often enhanced by adding hydrophilic functional groups to the hydrophobic polymers. This increases access of the released hydrolytic enzymes to the plastic surface (Orr et al., 2004; Tribedi and Sil, 2014; Nauendorf et al., 2016; Wilkes and Aristilde, 2017). Once attachment is initiated, subsequent degradation steps, such as biodeterioration, biofragmentation, assimilation, and mineralization can proceed more easily (Jaiswal et al., 2020).

The area of enzymatic PET hydrolysis may be progressed either by continuing to mine for new PET hydrolases with improved efficiencies, or through the application of protein engineering strategies to enhance the characteristics of enzymes that have already been identified (Liu et al., 2018). To date, rational design has proven useful in modifying cutinase enzymes to improve their activities. For example, site-directed mutagenesis, has been used to deliver more space or hydrophobicity in the Tfu_0883 active site (Silva et al., 2011) and helped to achieve 2.7- and 3-fold increases in activity for TfCut2 and Thc_Cut2, respectively (Herrero Acero et al., 2013; Wei et al., 2016).

In a study that focused on six key residues around the *Is*PETase substrate-binding pocket, Ma et al. (2018) created and tested for highly efficient mutants using rational protein engineering. The selected mutations aimed at optimizing the residues around the active site by replacing them with smaller amino acids to create space for the bulky aromatic groups of PET, or by substituting with more hydrophobic amino acids to give a greater affinity toward hydrophobic PET. Of the mutants that displayed improved activities compared to wild-type *Is*PETase, one variant (I179F) achieved a 2.5-fold increase in degradation of biaxially oriented PET film (35% crystallinity). This was determined based on weight loss of film after 48 h at 30°C (Ma et al., 2018; Kawai et al., 2019).

Following structural analysis, (Liu et al., 2018) designed a series of *Is*PETase mutants using site directed mutagenesis. Targets included key residues in the substrate-binding pockets and those which help stabilize active site rigidity. With mutations that led to increased aromaticity on the binding pocket edge or increased space at the binding center, higher activities were observed over the wild-type enzyme toward PET bottles. Activity was detected in terms of hydrolysis following 48 h incubation at 30°C and included a 3.1-fold increase by one mutant (Y87A) (Liu et al., 2018; Taniguchi et al., 2019).

In another study, a glycosylation strategy was employed to inhibit LC-cutinase native aggregation (Shirke et al., 2018). While LCC displays high thermostability (*T*_m_ = 86°C), initial thermal deactivation testing revealed a high tendency for aggregation by electrostatic interactions, limiting this enzyme in terms of stability and therefore, practical application. Glycosylated LCC (LCC-G), which was produced post-translationally by engineering three known *N*-glycosylation sites and expressing in *Pichia pastoris*, showed improvements in PET degradation under optimum temperature and concentration (Shirke et al., 2018).

Recently, Son et al. (2019) carried out a study to address the requirement for *Is*PETase variants with a highly increased capacity for PET hydrolysis and improved stabilities that enable the secreted enzyme to maintain its function in a harsh extracellular environment, over a long period at moderate temperatures. To achieve this, the group designed a plan for rational protein engineering based on the existing crystal structure of *Is*PETase. Following screening, an *Is*PETase (S121E/D186H/R280A) mutant displayed particularly enhanced characteristics (Son et al., 2019). This variant was designed such that subsite IIc was extended to provide a non-protruding, hydrophobic cleft, and a flexible β6–β7 connecting loop was stabilized (Joo et al., 2018). Upon comparison of the mutant with wild-type *Is*PETase, degradative activity toward PET film was shown to be 14-fold higher at 40°C. In addition, the melting temperature of this variant increased by 8.81°C and high thermostability was further verified by a heat-inactivation experiment (Son et al., 2019). The same group have since introduced mutations N246D and S242T, generating a quadruple *Is*PETase variant which displayed a 58-fold increase in hydrolyzing activity over its wild-type at 37°C, and maintained activity under these conditions for 20 days (Son et al., 2020).

Previous work demonstrated that the addition of the cation Ca^2+^ (or Mg^2+)^ had a positive effect on activating and stabilizing enzymes for PET hydrolysis (Kawai et al., 2014; Sulaiman et al., 2014; Miyakawa et al., 2015; Then et al., 2015). More recently, (Oda et al., 2018) investigated the role of three Ca^2+^ ions bound to separate sites (1–3) on Cut190 (S226P/R228S) and found that the ions had different roles in thermal and structural stabilization as well as enzyme activation, generating an overall change in protein conformation that help increase activity. Furthermore, by introducing a disulfide bond in site 2 for thermostability, the melting temperature was increased by at least 20–30°C when mutants were Ca^2+^-bound, with the bond mimicking the effect of Ca^2+^ (Oda et al., 2018).

Another group have demonstrated that pre-incubation of PET film with anionic surfactants attracted cationic *Is*PETase by helping to orient the enzyme active site with the hydrophobic PET surface (Furukawa et al., 2018). This accelerated enzymatic activity by 120-fold, with a 22% decrease in film thickness following incubation at 30°C for 36 h (Furukawa et al., 2018). In a follow-on study, they found that the activity of mutant TfCut2 (G62A/F209A) could be increased by the presence of a cationic surfactant. In this case, the enzyme has a negatively charged surface and is therefore brought closer to the PET film via electrostatic interactions upon addition of cationic surfactant (Furukawa et al., 2019). Moreover, when investigating the impact of different conditions on *Is*PETase, that may have relevance for industrial application, Liu et al. (2019) reported that organic solvents and detergents reduced the enzyme’s activity, while salts and glycerol led to enhanced enzymatic activity. Of the salts used, Na_2_SO_4_ had the most positive effect, which may be due to its higher effective cation concentration (Liu et al., 2019).

From an industrial perspective, wild-type microbial enzymes are usually modified to withstand the harsh and variable reaction conditions, and to satisfy the catalytic needs of a given process (Shukla, 2019). A synthetic biology approach combines a range of molecular and computational tools to generate enzymes that meet industrial expectations. Microbial metabolism can be manipulated through engineering of regulatory and genetic elements (e.g., promoter, terminator, and binding sequences), to optimize gene expression and protein production. Nanotechnology is another synthetic biology tool, that has been used for enzyme immobilization onto nanoparticle beads. This can lead to improvements in enzyme activity, stability, and thus reusability, as demonstrated by an immobilized lipase from a *Fusarium incarnatum* strain, which retained 75% of its degradation activity after 5 cycles when applied to treat waste cooking oil (Joshi et al., 2019). *In silico* studies and computer simulations bypass lab-scale investigations and are used, for example, to investigate enzyme-substrate interactions and substrate-binding efficiency (Shukla, 2019).

## Future Perspectives

### Bioremediation

Bioremediation refers to the use of living organisms or their enzymes to detoxify or restore contaminated sites, often by directing the natural capabilities of microbes toward environmental pollutants (Baba et al., 2016). Microorganisms have proven valuable in the remediation of environmental pollutants and various waste substances such as heavy metals (Kang et al., 2016; Igiri et al., 2018), polychlorinated biphenyls (PCBs) (Sharma J.K. et al., 2018; Elangovan et al., 2019) and even petroleum (Varjani, 2017; Koolivand et al., 2019). With respect to the contamination of marine and terrestrial environments by plastics, microplastics and their residues, it is vital to establish an efficient and carefully thought-out bioremediation strategy.

*Thermobifida* sp., *Fusarium* sp., *Saccharomonospora* sp., *Bacillus* sp., and *Pseudomonas* sp., are among the different microbial species that have to date been reported to degrade PET (Kumari and Chaudhary, 2020). Members of the *Pseudomonas* genus, which are noted for their extraordinary metabolic versatility, stand out as a natural degraders of synthetic substrates including different types of plastic and plastic associated monomers (Wierckx et al., 2015). In addition to hydrocarbons and other hydrophobic polymers, environmental *Pseudomonas* isolates have been shown to degrade polyethylene, polyethylene glycol, polypropylene, polyurethane, polystyrene, polyethylene succinate, polyvinyl alcohol, and polyvinyl chloride, to varying extents (Wilkes and Aristilde, 2017; Sharma, 2018). One microbial consortium, isolated from cow dung, consisting of thermophilic *Pseudomonas* sp., *Bacillus* sp., *Paenibacillus* sp., and *Stenotrophomonas* sp., was demonstrated to reduce the weight of high density polyethylene (HDPE) and low density polyethylene (LDPE) by 55 and 77%, respectively, following 120 days incubation (Skariyachan et al., 2017). Several *Pseudomonas* spp., including *Pseudomonas aeruginosa*, *Pseudomonas fluorescens*, *Pseudomonas protegens*, and *Pseudomonas chlororaphis*, have been found to be polyurethane degraders, despite the high durability of this material (Wilkes and Aristilde, 2017).

Microbes for plastic degradation, especially those which have been enhanced through genetic engineering, should not be released into nature without assessing the risk of introducing foreign microorganisms, that could potentially become invasive. Moreover, the microbes cannot be expected to simply “seek out” and degrade plastic in the environment as there are likely to be other, more easily accessible energy sources that they can consume (Jenkins et al., 2019). In the case of marine environments, where plastic waste most commonly accumulates, microorganisms for bioremediation may not be well adapted to operate under saltwater conditions. Therefore, the relevant enzymes may need to be produced in a better suited microbial cell factory (Moog et al., 2019). Another solution to facilitate bioremediation of ocean plastic is to use membrane-based systems featuring immobilized microbes or enzymes for plastic degradation. This would provide a “concentrated” source of plastic for the degradative microbes while also ensuring their containment (Jenkins et al., 2019).

Microorganisms are generally found living within complex communities that interact intensively and can adapt their overall metabolism to efficiently exploit the available resources (Jenkins et al., 2019). The use of a defined but flexible mixed microbial consortia (MCC) can help extend this concept to biotechnology processes and offers the advantages of inexpensive culture preparation and maintenance, reduced susceptibility to contamination, improved conversion efficiencies, and an alternative to improvement by genetic modification. Biocontainment measures which enable the termination of the MMC or certain strains, such as passcode or dead-man kill switches, are required for their use in natural environments (Jenkins et al., 2019). In this respect the aforementioned microbial consortium no. 46 from *I. sakaiensis*, may prove useful in bioremediation applications, either directly in the environment or in designated facilities for the treatment of PET waste and microplastics from marine and terrestrial sources (Hiraga et al., 2019). However, to fulfill the criteria for efficient bioremediation, natural microbial consortia require synthetic modifications to improve consortium interactions such as cell-cell signaling and metabolite exchange (Jaiswal et al., 2020).

Extensive research is still required for the bioremediation of PET-based environmental contamination and even with progress in this area, challenges will remain due to other types of synthetic polymers, present as waste in the environment. Although the potential of current candidates cannot be readily extended to large-scale bioremediation applications, the exploitation of their enzymes or metabolic pathways could be achieved through synthetic biology approaches and systemic biology, which utilizes multi-omics tools and biodegradation network databases, thus facilitating bioremediation of a complex substance like plastic (Jaiswal et al., 2020; Kumari and Chaudhary, 2020).

### Biological Recycling

Unlike glass or aluminum, where material can be repeatedly recovered in its original form, it is difficult to close the loop on the recycling of plastics. However, PET can to some extent be recycled using mechanical, chemical, or biological routes. In mechanical recycling, collected and sorted PET waste can be shredded and ground into powders before melting and reprocessing to other forms (Koshti et al., 2018). The resulting fibers, films and sheets cannot, however, be further recycled and are often incinerated, subsequently contributing to increased CO_2_ emissions. Chemical recycling aims to degrade PET into its basic monomers which can then be repolymerized. This method is unfavorable from an economic standpoint as the recovered monomers are more expensive than those from crude oil and despite its drawbacks, mechanical recycling is much more cost effective (Awaja and Pavel, 2005; Hiraga et al., 2019). In addition, chemical methods require the maintenance of high temperature and pressure as well as employing toxic reagents and several preceding unit operations (Wei and Zimmermann, 2017a; Koshti et al., 2018). For both chemical and mechanical recycling, the temperatures used are usually over 100°C and often exceed 200°C. Unfortunately, there are also several contaminants, that significantly interfere with the effectiveness of these PET recycling methods, whether they arise from other waste collected, the recycling process itself or misuse by consumers (Awaja and Pavel, 2005; Webb et al., 2013). Overall, PET recycling remains an expensive and inefficient process, regardless of the advantages over waste management via landfill and incineration, which are associated with major environmental drawbacks such as pollution and atmospheric release of harmful compounds (Zhang et al., 2004; Webb et al., 2013).

While chemical and mechanical processing of PET are currently in widespread use, biological recycling is emerging as a more sustainable solution as it can be performed under mild pH and comparatively low temperature conditions, without the use of hazardous chemicals (Awaja and Pavel, 2005; Webb et al., 2013; Wei and Zimmermann, 2017b). Biological recycling (or bio-recycling) is based on microbial catalysis of polymer bond cleavage reactions, which results in the recovery of monomers that can be subjected to further processing (Koshti et al., 2018). Bio-recycling holds great potential for improved efficiency and cost effectiveness over the aforementioned mechanical and chemical methods as well as helping to eliminate the occurrence of secondary pollutants that come with landfill and incineration (Webb et al., 2013). The possibility exists that a biological recycling process could be achieved using PET hydrolysis and further metabolism of its monomers by *I. sakaiensis* and other PET-degrading organisms and their enzymatic systems (Hiraga et al., 2019). However, bio-recycling is limited by the organism used, inherent polymer properties and the choice of pre-treatment. The future success of this process will rely on optimization and/or modification of these factors (Koshti et al., 2018). Given the time and variables associated with cultivating and maintaining microorganisms as well as extracting their enzymes and allowing the degradation reaction to proceed, biological recycling is relatively slow when compared with chemical or mechanical methods. Therefore, bio-recycling must currently be carried out in combination with at least one of these other approaches (Koshti et al., 2018; Farzi et al., 2019).

In one study, Farzi et al. (2019) assessed the biodegradation of PET by *Streptomyces* species in conjunction with a mechanical strategy. PET waste, in the form of bottles, was ground down to a powder form prior to the bacterial treatment, which comprised of 18 days incubation in a culture medium at 28°C. Powdered PET was separated by particle size (500, 420, 300, and 212 μm) with 50 mg samples of each size undergoing treatment. Through extraction of residual PET, final biodegradation percentages for the four groups were determined to be 49.2, 57.4, 62.4, and 68.8%, in order of decreasing particle size. Metabolites were analyzed via GC-MS (gas chromatography-mass spectrometry) to detect the hydrolysis products and to confirm the biodegradation process had taken place. In addition, a comparison between powdered and film sample degradation was undertaken using a PET film made from bottles, that was then subjected to the same biodegradation treatment, again for 18 days. Subsequent SEM analysis was used to visualize the surface of this film. While it was shown that degradation of the film surface did occur, results were not significant, particularly when compared to the powdered samples. It was evident from this lab-scale assessment that a mechanical powdering treatment can be very effective, giving high degradation efficiency (Farzi et al., 2019).

In another study, which combined bio-recycling with alkaline hydrolysis, an alkali-resistant whole cell biocatalyst was employed (Gong et al., 2018). Alkaline hydrolysis is one of several methods used to achieve PET chemical recycling (Carta et al., 2003; Koshti et al., 2018). A *Comamonas testosteroni* F4 strain that had previously been isolated from the wastewater of a factory producing PET (Zhang et al., 2004), was shown to degrade PET fibers and was subsequently subjected to evolutionary engineering to first give *C. testosteroni* F5, a strain which could utilize PET as a sole carbon source under alkaline conditions (Gong et al., 2012). Further modifications were completed to obtain the final alkali-tolerant strain, *C. testosteroni* F6. With whole-cell bacterial degradation of PET, the products do not accumulate in the culture as they are used during growth of the strain, thereby circumventing any feedback inhibition. Micro-sized PET particles (<10 μm) prepared by drying and grinding PET were used as the substrate, with no additional carbon sources. These particles were subjected to a 48 h fermentation at 37°C under three degradation conditions using: (1) the original F4 strain in a neutral medium; (2) the engineered F6 strain in an alkaline medium; (3) an alkaline medium without any bacterial culture. Under alkaline catalysis, the decomposition products were relatively simple, consisting mostly of TPA. Conversely, MHET, BHET, TPA, and methyl acrylate (MA) were the products obtained following degradation under biocatalytic conditions. The highest quantity of PET degradation products was observed under alkaline conditions with *C. testosteroni* F6, which was greater than the sum of both products under neutral conditions and alkaline hydrolysis (Gong et al., 2018).

Efficient biodegradation of highly crystallized PET was achieved by Chen et al. (2020) using a cell surface display-based strategy. An *Is*PETase-displaying yeast whole-cell biocatalyst was developed to overcome the low enzymatic activity of native *Is*PETase toward hc-PET, which limits its potential use in bio-recycling. Engineered *Pichia pastoris* yeast cells were constructed to functionally display *Is*PETase from a codon-optimized gene sequence. Endogenous glycosylphosphatidylinositol (GPI) proteins in the yeast cell wall were selected as anchor proteins, to which the enzyme could be connected via a flexible linker sequence (Chen et al., 2020). This strategy aimed to increase the probability of fully exposing the unique active-site cleft and substrate binding sites, which are crucial for *Is*PETase activity (Austin et al., 2018; Wu et al., 2018). A dramatic improvement in degradation efficiency was observed using this whole-cell biocatalyst system, with an approximated 36-fold increase in turnover rate compared with that of purified *Is*PETase. The whole-cell biocatalyst was shown to be robust and reusable, with the turnover rate remaining stable up to its seventh repeated use and under certain solvent/chemical conditions. In addition, the pH and thermostability of the enzyme increased following cell surface display. This approach presents a promising route for efficient bio-recycling and coupled with this, there is potential for activity to be further enhanced through similar cell-surface display of engineered *Is*PETase variants (Chen et al., 2020).

### The Role of PET Hydrolase Enzymes in a Circular Bioeconomy

Plastics are currently one of the major challenges in establishing a circular economy. The aim of a circular economy is to promote sustainability and efficiency by creating loops which feed resources back into the economy to make the same or new products. Plastic production is relatively sustainable when compared with the resource-intensive processes associated with other materials such as glass and metal. That said, there is a huge need to re-evaluate plastic design, production, use and management in order to improve its ability to re-enter the system and to ensure that maximum value is being recovered (Hahladakis et al., 2020). Through a combination of biodegradation and biosynthesis, there is potential for a circular, bio-based PET economy that could play a significant role in reducing the associated detrimental environmental effects. In general, the low production cost of plastic has played a major role in accelerating its mismanagement as reuse does not offer an economic advantage. While current strategies remain the most profitable option, a PET bioeconomy could match public expectations for alternative PET management, which is already leading to progress in terms of policies and regulations. In this way, the financial gap between strategies could be partially bridged, enabling an opportunity for further development of sustainable PET processing and therefore improving the environmental impact and rates of recycling in the longer term (Salvador et al., 2019).

In this respect, a strategy for movement toward a circularized PET economy which employs PET hydrolase enzymes to establish PET as a biotechnological feedstock has been proposed (Salvador et al., 2019). Due to the “bow-tie” metabolic structure associated with microbes, there is great potential for biodegradation products to be transformed into a range of useful molecules by channeling the monomers into central metabolism and linking to relevant biosynthetic pathways. This could be achieved by using metabolic engineering and synthetic biology to incorporate PET hydrolase enzymes into a microbial biosynthesis chassis, thereby helping to create revenue from PET waste while also reducing its release into the environment (Salvador et al., 2019; Taniguchi et al., 2019; Blank et al., 2020). Recently, the photosynthetic microalga *Phaeodactylum tricornutum* was employed as a chassis to produce and secrete an engineered *Is*PETase which displayed degradative activity toward different PET substrates (Moog et al., 2019). These cell factories could be valuable in future applications, including the design of photobioreactors capable of PET biodegradation and synthesis of new PET from the resulting monomers. To date *E. coli* and *B. subtilis* are among other microbial systems that have been successfully utilized for the creation of synthetic *Is*PETase cell factories (Huang et al., 2018; Moog et al., 2019; Seo et al., 2019).

Prior to central metabolism, TPA is converted into protocatechuic acid (PCA) that can be further degraded via several different pathways/routes (Frazee et al., 1993; Maruyama et al., 2004; Kasai et al., 2009). This is important to consider when developing PET-based bioprocesses because different pathways will generate a distinct range of metabolites, with varied applications. For example, PCA itself has been used to synthesize adipic acid, which is an industrially relevant metabolite (Johnson et al., 2016; Salvador et al., 2019) and EG, which can be degraded into acetate or glyoxylate (GLA), has an even more diverse metabolism than TPA (Child and Willetts, 1978; Kataoka et al., 2001; Trifunović et al., 2016). Significantly, EG has been transformed into the bioplastic polyhydroxyalkanoate (PHA) by an engineered *Pseudomonas putida* KT2440 strain, presenting an opportunity to use PET monomers as a feedstock for the production of a biodegradable alternative (Franden et al., 2018; Blank et al., 2020).

Bio-PET, which refers to a PET polymer that is at least partially derived from biological sources, can be produced through the microbial synthesis of TPA and EG. By cutting down the dependence on fossil fuel-derived “virgin” PET, this method could make a significant contribution to a sustainable and circular PET economy (Salvador et al., 2019). While biosynthesis of aromatic compounds by microbes has not developed to the same extent as their degradation, it has been proposed to produce terephthalic acid by harnessing the shikimate pathway to provide *p*-toluate which could be transformed into TPA (Osterhout et al., 2014). It may also be possible to achieve sustainable TPA biosynthesis by using aromatics from renewable sources like lignin. However, many complexities are associated with biological TPA production and therefore, it is only EG that is currently produced biologically from renewable feedstocks to give bio-PET (Salvador et al., 2019). This has been achieved by engineering artificial pathways into microorganisms, enabling the use of renewable plant feedstocks, such as xylose (Cam et al., 2016) and corn stalk (Pang et al., 2011).

Most recently, the aforementioned LC-cutinase enzyme was engineered with the aim of maximizing catalysis of PET depolymerization and was subsequently shown to give 90% conversion within just 10 h. New PET was successfully synthesized from the TPA monomers obtained (Tournier et al., 2020). LCC was demonstrated to outperform four other PET hydrolytic enzymes, *Is*PETase, *T. fusca* hydrolases TfH (BTA-1) and BTA-2, and *F. solani* cutinase when compared in terms of specific depolymerization rate of pre-treated amorphous PET film at 65°C. Then, following computational identification of amino acid residues for site-saturation mutagenesis, a range of variants were generated with the aim of introducing enhanced thermostability and specific activity toward bottle-grade PET. Four quadruple variants, ICCG, ICCM, WCCG, and WCCM, were ultimately selected for further evaluation (Tournier et al., 2020).

These engineered enzymes were assessed under bioreactor conditions for degradation of post-consumer colored-flake PET (PcW-PET) waste. This is the residue that remains once clear PET waste for mechanical recycling has been removed (Tournier et al., 2020). PcW-PET was pre-treated using extrusion and micronization processes that are employed widely in plastics industries (Awaja and Pavel, 2005; Barboza Neto et al., 2014). The reaction temperature was set at 72°C to maximize kinetic turnover. WCCG and ICCG achieved the best conversion levels, with 85% and 82% conversion within 15 h and 20 h, respectively. Wild-type LCC reached only 53% conversion in 20 h, owing to its lower thermostability that rapidly reduces the reaction kinetics after just 2 h. Following subsequent comparison at 3 milligrams of enzyme per gram of PET, WCCG and ICCG demonstrated 90% depolymerization after 10.5 h and 9.3 h, respectively. This was further increased to a 150 L pilot-scale process using high content of PcW-PET (200 g/kg), which utilized 2 mg_enzyme_/g_PET_ in a trade-off between enzyme cost and productivity (Tournier et al., 2020).

Initial process development investigated the recycling of terephthalic acid (TPA) only as this is the main component of PET by weight, with 1 ton of PET waste leading to 863 kg of TPA. Terephthalate monomers were purified to a level exceeding 99.8% through the use of industrially applied methods such as discoloration by activated carbon and crystallization (Meyer, 1966; Mohammad-Khah and Ansari, 2009). TPA monomers were used in the synthesis of “virgin” PET, which required three consecutive steps: esterification, polycondensation and solid-state polymerization. The resulting PET was used to blow new bottles, which exhibited improved lightness and similar mechanical characteristics to commercially available PET bottles, thus creating a closed-loop recycling process (Tournier et al., 2020).

Biological strategies, even in conjunction with established and emerging initiatives to curtail plastic pollution, both voluntary and regulatory, will not meet the current requirements. Instead, a combination of several different multi-tiered approaches will be essential in eliminating this issue (da Costa et al., 2020). The matter has been further complicated by the COVID-19 pandemic, whereby plastic-based personal protective equipment, such as masks and gloves, along with single-use plastics due to hygiene concerns and food packaging demands has resulted in delays in enforcing plastic reduction policies (Patrício Silva et al., 2020).

**FIGURE 5:**
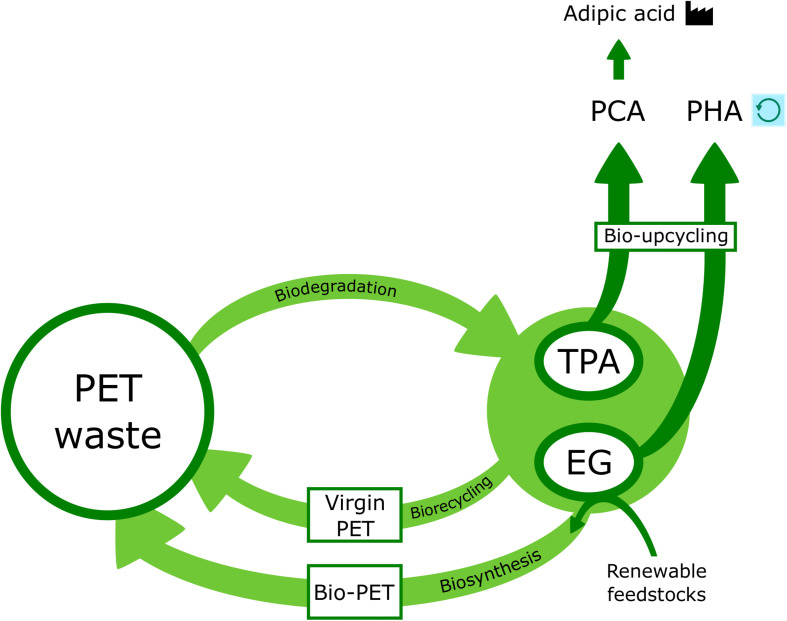
Prospective overview of PET in the circular economy. Monomers resulting from biodegradation of PET may be recovered for manufacture of virgin PET (biorecycling) or combined with microbially synthesized monomers for a bio-based alternative (biosynthesis). Adipic acid, an important chemical precursor used primarily in nylon production, and polyhydroxyalkanoates (PHAs), which are both bio-based and biodegradable, are among the value-added products that may be obtained through transformation of TPA and EG monomers (bio-upcycling).

Overall, the prospects for establishing a PET circular bioeconomy are still promising, with several strategies underway that use PET hydrolytic enzymes (or whole cells) to give TPA and EG for synthesis of fresh PET or for conversion into value-added products. Biodegradation-based processes could be complemented by the sustainable synthesis of TPA and EG monomers for bio-PET. Some of the potential routes for PET in the circular economy are summarized (Figure 5).

## Final Remarks

While the efforts to harness the power of microbial PET hydrolase enzymes are ongoing, research in this area has been encouraging and new knowledge has quickly accumulated. Many different groups have been working to identify and characterize PET hydrolyzing microorganisms and their enzymes to explore degradative activities, connect structure with function and uncover the mechanisms behind microbial processing of synthetic polymers. This work, together with the use of powerful enzyme technologies to create improved variants, has already improved the feasibility of practical PET degradation and will continue to underpin future applications as well as process optimization. Some of the most promising cases highlighted in this review have included pilot-scale PET hydrolysis using engineered LC-cutinase together with *I. sakaiensis* enzymes, various *Is*PETase mutants and *IsPETase* cell factories or display systems that may enable the break down of high crystallinity PET under mild reaction conditions. Although enzymatic systems that resemble the *I. sakaiensis* PETase-MHETase PET processing pair have not yet been observed in any other bacterial genome sequences, the chances of finding potential PET metabolic enzymes in the future is likely to increase with the aforementioned ongoing rapid expansion of genomic and metagenomic sequence data.

With the aim of addressing different types of PET waste, especially high crystallinity forms, there is a need for further investigation into strategies such as synergistic combined enzyme systems, use of ionic surfactants, circumvention of PET intermediate inhibition and the development of biosynthetic cell factories. It would also be useful to establish standard protocols and PET substrates to allow for more meaningful comparisons when assessing PET hydrolase candidates and determining optimal process conditions. The development of microbes and enzymes for biological recycling will help progress their implementation in other areas, for instance in bioremediation strategies. Despite the improvements that can be accomplished using enzyme engineering, “mining” for new PET hydrolases is still vital to gain a better understanding of plastic degradation in nature and to identify inherent activities that can be enhanced to enable treatment of PET waste *in situ*. Culture based screening strategies coupled with metagenomic based approaches targeting various PET or hydrocarbon contaminated environmental samples is likely to uncover even better evolved microbial activities for PET metabolism.

In recent years there has been a significant shift in awareness and concern surrounding plastic-related environmental issues. For the most part, plastic is not inherently “bad,” with alternative materials often having a higher overall impact when factors such as production requirements and bulk shipping are considered. Plastic packaging also provides the current best solution in niche sectors, for example, in helping prevent food spoilage in the food industry. Today’s plastic problem is rooted in a loss of control and the inability of waste collection, disposal, and recycling infrastructure to match the pace of the plastics industry. Therefore, a multi-faceted approach is essential in addressing the complexities of this issue. In the context of contributing microbial based solutions, there must be a balance between process optimization and environmental consequences, such that counterproductivity is avoided. A collaborative effort is needed to reimagine the life cycle of PET, where its value is maximized, and it is treated as a feedstock rather than as a waste product after use. The development of systems which employ microbes for PET biodegradation and biosynthesis hold the potential to fulfill these requirements, at least partially. This shift from a linear to circularized system of PET production and processing could help meet public demands by reducing the need to “tap into” fossil fuel reserves and incentivizing better handling of PET waste.

This review serves as an important reminder to other researchers in this field, and to the wider scientific community, of the numerous PET hydrolase studies that have been carried out to date, with an emphasis on the most recent examples and advances. By highlighting the major challenges in studying and implementing enzymes for PET degradation, a clearer consensus can be developed to guide future work. Plastic pollution has become a global concern, and the interest in sustainable change is shared by the general public, environmental organizations, and by governments and industrial leaders. Therefore, it is hoped that the different perspectives presented in this review will play a role in helping to encourage a more multi-disciplinary approach to address the ongoing problem of plastic waste streams.

## Author Contributions

CMC wrote the manuscript. ADWD and DJC supervised and corrected the manuscript. All authors read and approved the final manuscript.

## Conflict of Interest

The authors declare that the research was conducted in the absence of any commercial or financial relationships that could be construed as a potential conflict of interest.
